# Notes and Notices

**Published:** 1894-04

**Authors:** 


### NOTES AND NOTICES.

ANNUAL RE-UNION OF THE ALUMNI ASSOCIATION OF THE HAHNEMANN MEDICAL COLLEGE, PHILADELPHIA, TUESDAY, MAY 8TH, 1894.—The Alumni Association of the Hahnemann Medical College, Philadelphia, requests the pleasure of the company of the Alumni of the College, at its Annual Re-union and Banquet, on Tuesday, May 8th, 1894.

The Business Meeting will convene at 4.30 P. M. in Alumni Hall, Hahnemann Medical College, Broad Street above Race, Philadelphia, and the Banquet will be held at 10 P. M. at "The Stratford," corner of Broad and Walnut Streets.

The Trustees and Faculty of the College extend a cordial invitation to all the members of the Alumni and their friends to attend the Forty-sixth Annual Commencement, to be held on the same evening, at 8 o'clock, at the Academy of Music, Broad and Locust Streets, Philadelphia.

Banquet cards can be secured from any officer of the Association at $3.50 each. The cards being limited to two hundred, the Committee cannot guarantee to furnish any applied for after May 7th, 1894. If you can make arrangements to be present at the Banquet, notify the Secretary and he will secure a place for you. W. W. VAN BAUN, M. D., Secretary, 419 Pine Street, Philadelphia, Pa.

FREDERICK STEARNS & CO., of Detroit, Mich., have issued an ornamental blotter in colors. The colors are produced by the same process as was their calendar noticed in the February issue of this journal at page 64. They will be furnished free to any physician who makes application.

DR. MILLIE J. CHAPMAN desires to announce the removal of her office from 916 Penn Avenue to 804 Penn Avenue. Hours, 9 to 11 A. M., 2 to 4 P. M. Residence, No. 5824 Rural Avenue, Pittsburg, Pa. Hours, 6 to 8 P. M.

JOHN M. SCUDDER, M. D., editor of *The Eclectic Medical Journal*, died on February 17th, 1894. Hereafter the medical publishing business, established by him will be conducted, as during the past three years, by his sons, but under the new firm name of John M. Scudder's Sons.

*The Eclectic Medical Journal*, established in 1833, will be edited by John K. Scudder, M. D. A complete line of Eclectic Medical Books, our specialty, always on hand. All communications in reference to *The Eclectic Medical Journal*, either subscriptions or advertisements, and all orders for books should be addressed, and remittances made payable to John M. Scudder's Sons, No. 228 West Court Street, Cincinnati, Ohio.

THE NORTH INDIANA AND SOUTHERN MICHIGAN HOMŒOPATHIC MEDICAL ASSOCIATION will hold its sixth semi-annual meeting in the Century Club Ro m, 115 Main Street, Elkhart, Indiana, Thursday, May 3d, 1894. For further information, address Dr. H. A. Mumaw, Secretary, Elkhart, Indiana.

